# Proangiogenic Effect of Metformin in Endothelial Cells Is via Upregulation of VEGFR1/2 and Their Signaling under Hyperglycemia-Hypoxia

**DOI:** 10.3390/ijms19010293

**Published:** 2018-01-19

**Authors:** Sherin Bakhashab, Farid Ahmed, Hans-Juergen Schulten, Fahad W. Ahmed, Michael Glanville, Mohammed H. Al-Qahtani, Jolanta U. Weaver

**Affiliations:** 1 Biochemistry Department, King Abdulaziz University, Jeddah P.O. Box 80218, Saudi Arabia; sbakhashab@kau.edu.sa; 2 Institute of Cellular Medicine, Newcastle University, Newcastle Upon Tyne NE2 4HH, UK; nfahadahmed@gmail.com (F.W.A.); michael.glanville@newcastle.ac.uk (M.G.); 3 Centre of Excellence in Genomic Medicine Research, King Abdulaziz University, Jeddah P.O. Box 80216, Saudi Arabia; fahmed1@kau.edu.sa (F.A.); hschulten@kau.edu.sa (H.-J.S.); mhalqahtani@kau.edu.sa (M.H.A.-Q.); 4 Queen Elizabeth Hospital, Gateshead, Newcastle Upon Tyne NE9 6SH, UK; 5 Cardiovascular Research Centre, Newcastle University, Newcastle Upon Tyne NE2 4HH, UK

**Keywords:** cardiovascular disease, ischemia, diabetes, VEGF signaling

## Abstract

Cardiovascular disease is the leading cause of morbidity/mortality worldwide. Metformin is the first therapy offering cardioprotection in type 2 diabetes and non-diabetic animals with unknown mechanism. We have shown that metformin improves angiogenesis via affecting expression of growth factors/angiogenic inhibitors in CD34^+^ cells under hyperglycemia-hypoxia. Now we studied the direct effect of physiological dose of metformin on human umbilical vein endothelial cells (HUVEC) under conditions mimicking hypoxia-hyperglycemia. HUVEC migration and apoptosis were studied after induction with euglycemia or hyperglycemia and/or CoCl_2_ induced hypoxia in the presence or absence of metformin. HUVEC mRNA was assayed by whole transcript microarrays. Genes were confirmed by qRT-PCR, proteins by western blot, ELISA or flow cytometry. Metformin promoted HUVEC migration and inhibited apoptosis via upregulation of vascular endothelial growth factor (VEGF) receptors (VEGFR1/R2), fatty acid binding protein 4 (*FABP4*), ERK/mitogen-activated protein kinase signaling, chemokine ligand 8, lymphocyte antigen 96, Rho kinase 1 (*ROCK1*), matrix metalloproteinase 16 (*MMP16*) and tissue factor inhibitor-2 under hyperglycemia-chemical hypoxia. Therefore, metformin’s dual effect in hyperglycemia-chemical hypoxia is mediated by direct effect on VEGFR1/R2 leading to activation of cell migration through *MMP16* and *ROCK1* upregulation, and inhibition of apoptosis by increase in phospho-ERK1/2 and *FABP4*, components of VEGF signaling cascades.

## 1. Introduction

Cardiovascular disease (CVD) remains the leading cause of death worldwide according to the World Health Organization 2016 mortality database. The outcome of CVD management is affected by diabetes mellitus (DM), which results in a two- to four-fold increased risk of CVD [1,2]. The outcomes of CVD interventions such as percutaneous coronary intervention (PCI) and coronary artery bypass graft are much worse in diabetic than non-diabetic individuals [3,4].

Diabetes associated endothelial dysfunction is a known early step in the adverse sequence of events leading to the development of micro- and macrovascular complications, which results in mortality linked to diabetes in 75% of cases [5]. Vascular complications include both qualitative and quantitative changes in vascular architecture, in particular: abrogation of neovascularization and remodeling of the existing vasculature that results in a lack of ability to control ischemic injury. Understanding the mechanisms involved in diabetes associated impaired vascular repair and known interventions is paramount. Therefore, investigating drugs affecting angiogenesis during a therapeutic window for myocardial infarction (1 h), delayed (3 h) and prolonged (12 h) period of ischemia and acute hyperglycemia is of importance. Metformin is the first hypoglycemic therapy to display cardioprotective properties as shown in long-term randomized clinical trials [6,7]. Furthermore, when used in patients with metabolic syndrome, post-PCI myocardial injury was improved [8].

Pre-treatment of diabetic patients with metformin was found to be associated with reduced myocardial infarction (MI) size compared to non-metformin treated [9]. Additionally, studies in animal models suggested that metformin at physiological doses improved coronary blood flow following MI [10], and limited infarct size in diabetic rats [11]. Our recent work has shown that metformin improved angiogenesis by augmenting the expression of VEGFA and reducing angiogenic inhibitors in CD34^+^ cells under hyperglycemia-hypoxia [12]. However, no data on the direct effect of physiological doses of metformin on vascular repair (migration, apoptosis) under hyperglycemia-hypoxia is available to date.

In this paper, we hypothesized that metformin at physiological dose enhances endothelial cell (EC) survival and migration under the conditions of hyperglycemia and hypoxia. As it is important for the research to be of translational value, we specifically applied clinically relevant physiological doses of metformin. Although cell culture systems have certain limitations in mimicking and simulating CVD; they are indispensable tools to unravel critical underlying molecular mechanisms of the disease. Therefore, we employed freshly isolated HUVEC from different donors as an in vitro model to study the effect of physiological concentration of metformin on cell survival, migration and gene expression under chemical hypoxia and hyperglycemia.

## 2. Results

### 2.1. Metformin Promotes Endothelial Cell Migration under Hyperglycemia-CoCl_2_

**Figure 1:**
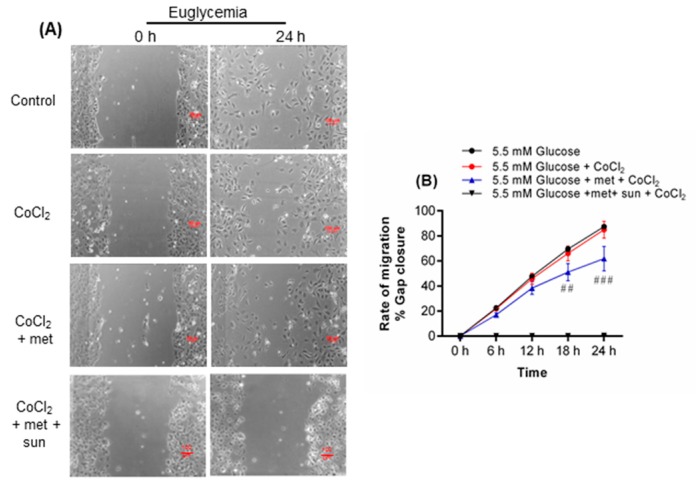
Metformin impairs cell migration in HUVEC exposed to euglycemia and CoCl_2_. (**A**) HUVEC were incubated for 24 h with euglycemia in the presence or absence of metformin. Scratch lines were created on confluent monolayers. The media containing different glucose concentrations and metformin were replaced. Then cells were incubated with CoCl_2_ for 24 h in a 5% CO_2_ chamber that was connected to CCD camera. Images were acquired every hour, and three independent biological experiments were performed at which each condition was assessed in duplicate. The scratch area was measured using NIS Elements software. (**B**) CoCl_2_ induction exhibited no significant effect on cell migration under euglycemia, whereas metformin reduced migration after 18 h. Sunitinib (0.1 µmol/L) was used as a negative control, therefore the line with sunitinab is on *x* axis as cell migration not affected. Results are expressed as mean ± SEM and were analyzed by one-way ANOVA followed by LSD, ^##^ *p* < 0.01, ^###^ *p* < 0.001 compared pairwise, i.e., the metformin-treated versus metformin-untreated condition. Scale bar is 100 µm. Key: met: metformin; sun: sunitinib.

**Figure 2:**
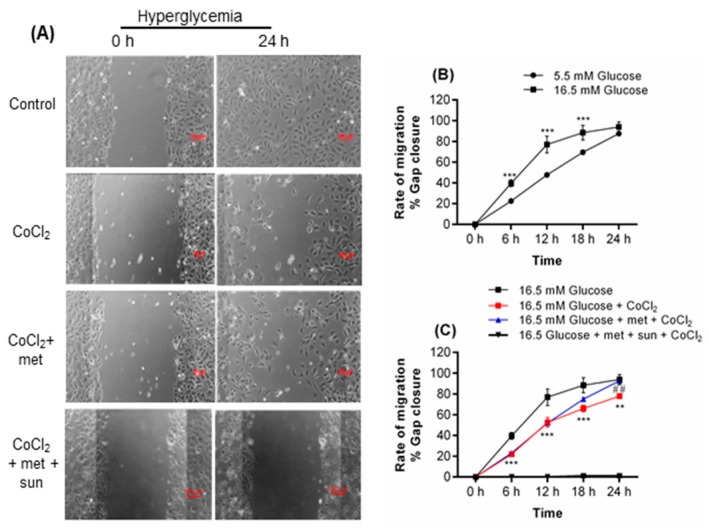
Metformin improves cell migration in HUVEC exposed to hyperglycemia and CoCl_2_. (**A**) HUVEC were incubated for 24 h with hyperglycemia in the presence or absence of metformin. Scratch lines were created on confluent monolayers. The media containing different glucose concentrations and metformin were replaced. Then cells were incubated with CoCl_2_ for 24 h in a 5% CO_2_ chamber that was connected to CCD camera. Images were acquired every hour, and three independent biological experiments were performed at which each condition was assessed in duplicate. The scratch area was measured using NIS Elements software. (**B**) Hyperglycemia increased migration after 6, 12, and 18 h; (**C**) whereas hyperglycemia-CoCl_2_ significantly reduced migration. Metformin increased cell migration under hyperglycemia-CoCl_2_. Sunitinib was used as a negative control, therefore the line with sunitinab is on *x* axis as cell migration not affected. Results are expressed as mean ± SEM and were analyzed by one-way ANOVA followed by LSD, ** *p* < 0.01, *** *p* < 0.001 compared to the control. ^##^ *p* < 0.01, ^###^ *p* < 0.001 compared pairwise, i.e., the metformin-treated versus metformin-untreated condition. Scale bar is 100 µm. Key: met: metformin; sun: sunitinib.

Metformin reduced cell migration under euglycemia-CoCl_2_ at 18 (*p* < 0.01) and 24 h (*p* < 0.001, Figure 1B) of CoCl_2_ but accelerated cell migration under hyperglycemia-CoCl_2_ at 24 h of CoCl_2_ (*p* < 0.01, Figure 2C).

Euglycemia-CoCl_2_ had no effect on EC migration compared to control (Figure 1A,B). However, hyperglycemia alone significantly increased migration at 6 (*p* < 0.001), 12 (*p* < 0.001), and 18 h (*p* < 0.001, Figure 2A,B) but not at 24 h (plateau phase). Hyperglycemia-CoCl_2_ inhibited migration at 6 (*p* < 0.001), 12 (*p* < 0.001), 18 (p < 0.001), and 24 h of CoCl_2_ (*p* < 0.01, Figure 2A,C) compared to hyperglycemia alone.

### 2.2. Inhibition of Metformin Action Mediated by VEGFA in Cell Migration Assay

Sunitinib, a VEGF inhibitor, blocked the effect of metformin in euglycaemia-CoCl_2_ and hyperglycemia-CoCl_2_ (Figure 1A,B and Figure 2A,C).

### 2.3. Metformin Reduces Apoptosis under Hyperglycemia-CoCl_2_

**Figure 3:**
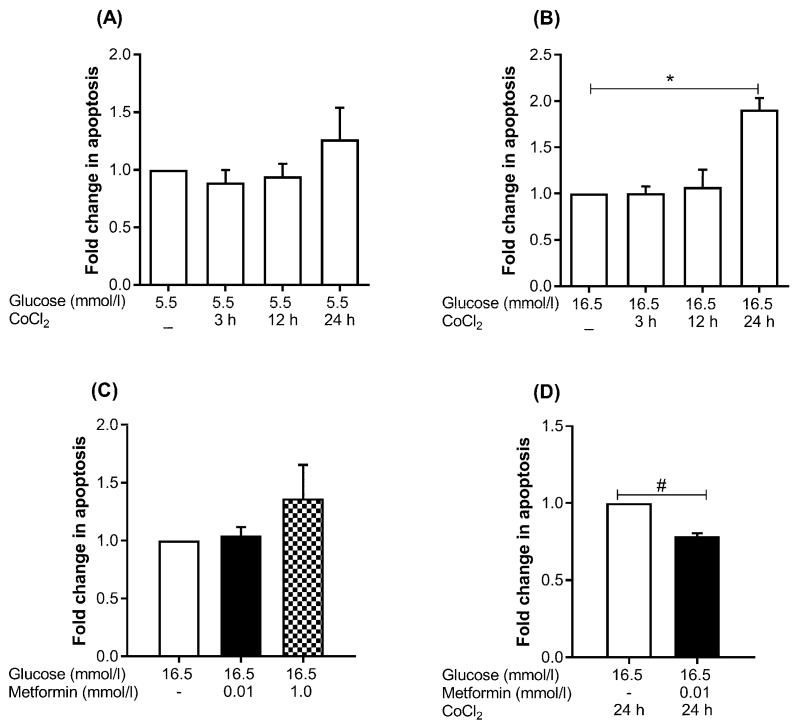
Metformin improves cell survival with hyperglycemia-CoCl_2_. (**A**) HUVEC were treated with 5.5 or (**B**) 16.5 mmol/L glucose for 48 h exposed to chemical hypoxia for 3, 12 or 24 h in the absence of metformin, (**C**) hyperglycemia treated with metformin (0.01 mmol/L) and supra-physiological concentration of metformin (1.0 mmol/L), and (**D**) hyperglycemia exposed to CoCl_2_ for 24 h and parallel cultures were treated with metformin. Apoptosis was assessed by Annexin V staining and flow cytometry. Results are representative of 3 independent experiments and expressed as mean ± SEM and were analyzed by paired *t*-test, * *p* < 0.05, compared to hyperglycemia, ^#^ *p* < 0.05 compared pairwise, i.e., metformin-treated condition versus untreated condition.

Metformin significantly reduced the apoptosis (−1.3-fold, *p* = 0.045) under hyperglycemia-CoCl_2_ at 24 h of CoCl_2_ (Figure 3D). However, metformin concentration exhibited no effect on HUVEC apoptosis under euglycemia-CoCl_2_ at 3, 12, or 24 h of CoCl_2_ or under hyperglycemia-CoCl_2_ at 3 and 12 h of CoCl_2_ (Figure S1). A supra-physiological concentration of metformin (1.0 mmol/L) non-significantly enhanced apoptosis (1.3-fold, *p* > 0.05) in HUVEC treated with hyperglycemia for 48 h (Figure 3C).

No effect on apoptosis at 3 and 12 h of CoCl_2_ was detected neither under euglycemia nor hyperglycemia (Figure 3A,B). At 24 h of CoCl_2_ there was a non-significant increase (1.3-fold, *p* > 0.05) in the apoptotic cells under euglycemic conditions (Figure 3A); but a significant increase (1.9-fold, *p* = 0.018) under hyperglycemia-CoCl_2_ (Figure 3B).

### 2.4. Microarray Gene Expression Profiling in HUVEC and Western Blot

In the microarray experiments, the number of differentially expressed genes with a *p*-value < 0.05 and a cutoff fold change of 1.5 varied considerably between the conditions.

### 2.5. Metformin Has No Effect on HIF-1α Gene/Protein Expression under Hyperglycemia-CoCl_2_

Transcriptome analysis demonstrated that physiological concentration of metformin exerted no effect on HIF-1α signaling when HUVEC were exposed to CoCl_2_ for 1, 3, 12 h either under euglycemia or hyperglycemia. Western blot (WB) assay revealed that metformin led to a significant downregulation of HIF-1α protein levels at 1 h of CoCl_2_ under euglycemia-CoCl_2_ (−7.6-fold, *p* < 0.001) but exhibited no effect under hyperglycemia-CoCl_2_ (Figure S2).

HIF-1α mRNA levels at 1 and 3 h of CoCl_2_ were unchanged under euglycemia-CoCl_2_ and hyperglycemia-CoCl_2_. Whereas, a significant decrease was detected at 12 h of CoCl_2_ in both euglycemia (−1.6-fold, *p* = 1.4 ×10^−6^) and hyperglycemia (−1.9-fold, *p* = 4.4 × 10^−9^). WB revealed a significant increase in HIF-1α protein at 1 h of CoCl_2_ under euglycemia-CoCl_2_ (3.4-fold, *p* < 0.01) but no significant increase under hyperglycemia-CoCl_2_ (Figure S2).

### 2.6. Metformin Has No Significant Effect on VEGFA Gene Expression but Increased Protein Expression at 1 h of CoCl_2_ under Hyperglycemia-CoCl_2_

**Figure 4:**
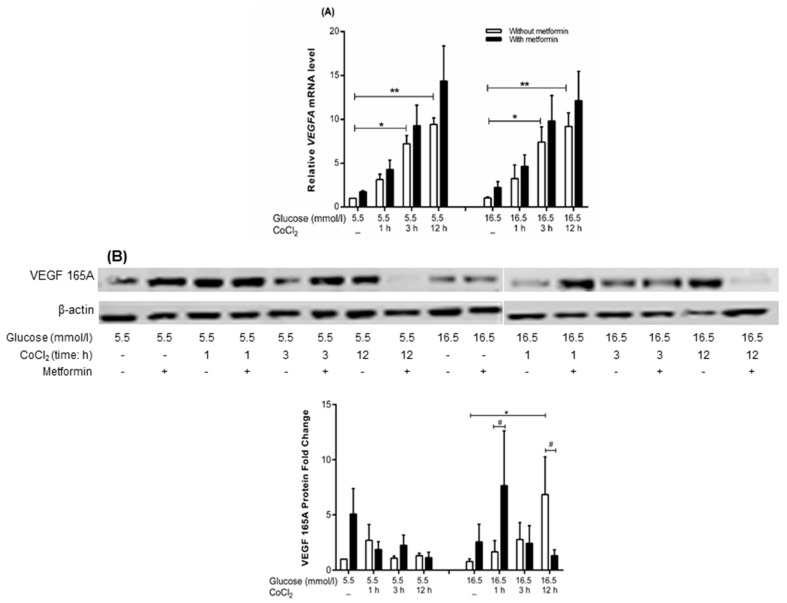
Effect of metformin on VEGFA mRNA and VEGF 165A protein under euglycemia-CoCl_2_, hyperglycemia and hyperglycemia-CoCl_2_. HUVEC were treated with hyperglycemic or euglycemic glucose concentrations as a control. After 24 h, metformin was added to euglycemic and hyperglycemic cultures, then CoCl_2_ was added for either 1, 3 or 12 h. (**A**) The variation in mRNA expression levels of VEGFA was assessed by qRT-PCR on three independent biological replicates and (**B**) the variation in protein levels of VEGF 165A was assessed by western blot on three independent biological replicates. Results are presented as mean ± SEM and were analyzed using one-way ANOVA followed by LSD, * *p* < 0.05, ** p < 0.01 compared to the control. *^#^ p* < 0.05 compared pairwise, i.e.; metformin-treated condition versus untreated condition.

Metformin exhibited no effect on *VEGFA* mRNA expression under euglycemia-CoCl_2_ and hyperglycemia-CoCl_2_ up to 12 h as assayed by microarray and qRT-PCR (Figure 4A) versus parallel condition without metformin.

In WB, metformin exhibited no significant effect on VEGF 165A protein expression under euglycemia-CoCl_2_. However, under hyperglycemia-CoCl_2_, metformin significantly increased VEGF 165A protein level at 1 h of CoCl_2_ (7.7-fold, *p* = 0.012), whereas the level decreased at 12 h of CoCl_2_ (−5.2-fold, *p* = 0.019) versus parallel conditions without metformin (Figure 4B).

Transcriptome analysis showed a time dependent increase in *VEGFA* mRNA upon exposure to CoCl_2_ (1, 3, or 12 h) under euglycemia (1.8-fold, 3.79-fold, and 5.15-fold respectively) and hyperglycemia (1.6-fold, 3.5-fold, 4.7-fold respectively). qRT-PCR validation showed upregulation of *VEGFA* upon exposure to CoCl_2_ at 3 and 12 h under euglycemia-CoCl_2_ (7.2-fold, *p* = 0.017 and 9.4-fold, *p* = 0.002 respectively versus no CoCl_2_), and hyperglycemia-CoCl_2_ (7.4-fold, *p* = 0.015 and 8.4-fold, *p* = 0.005 respectively versus hyperglycemia alone) as illustrated in Figure 4A.

In WB VEGF 165A protein expression was unchanged under euglycemia-CoCl_2_ but was significantly upregulated at 12 h of CoCl_2_ under hyperglycemia-CoCl_2_ (6.8-fold, *p* = 0.011 compared to hyperglycemia alone) but not at 1 or 3 h (Figure 4B).

### 2.7. Metformin Activates VEGF Downstream Signaling

Several key signaling cascades, including ERK/MAPK and PI3K/Akt pathways are known to be implicated in the modulation of VEGF induced angiogenic and proliferation effects mediated via VEGFR1 [13]. Microarray studies showed that the mRNA of VEGFR1 (alias FLT1) was upregulated (1.7-fold, *p* = 7.36 × 10^−3^) under euglycemia-CoCl_2_ at 12 h of CoCl_2_ but not under hyperglycemia-CoCl_2_. A number of VEGFR1 downstream genes was significantly differentially expressed under euglycemia and hyperglycemia following 12 h of CoCl_2_ exposure. Among these genes was the mitogen-activated protein kinase kinase 1 (*MAP2K1*), a key molecule in the ERK1/2 MAP kinase pathway, with 1.7-fold (*p* = 5.37 × 10^−9^) upregulated under euglycemia-CoCl_2_, and 1.6-fold (*p* = 6.8 × 10^−8^) upregulated under hyperglycemia-CoCl_2_. Moreover, gene expression of phosphoinositide-3-kinase, regulatory subunit 1 (*PI3KR1*) which is a PI3K/Akt signaling molecule, was increased at 12 h CoCl_2_ with euglycemia to 1.9-fold (*p* = 2.25 × 10^−7^) and hyperglycemia to 1.7-fold (*p* = 7.37 × 10^−6^). In contrast, *PI3KR3* was detected to be downregulated by −2.5-fold (*p* = 2.09 × 10^−9^) under euglycemia-CoCl_2_ at 12 h of CoCl_2_.

To substantiate these findings, the protein levels of VEGFR1 and VEGFR2 were assessed by ELISA. Metformin increased the expression of VEGFR1 and VEGFR2 only under hyperglycemia-CoCl_2_ at 3 h of CoCl_2_ (1.5-fold, *p* = 0.02 and 1.4-fold, *p* = 0.02 respectively (Figures S4 and S5)).

The expression of VEGFR1 was upregulated under hyperglycemia-CoCl_2_ at 1, 3, 12 h of CoCl_2_ exposure (1.8-fold, *p* = 0.02; 1.6-fold, *p* = 0.04; 1.9-fold, *p* = 0.01) compared to hyperglycemia alone (Figure S3a). Whereas, the expression of VEGFR2 showed downregulation under euglycemia-CoCl_2_ at 1, 3, 12 h of CoCl_2_ (−1.3-fold, *p* = 0.034; −1.3-fold, *p* = 0.04; −1.9-fold, *p* < 0.001) and hyperglycemia (−1.4-fold, *p* = 0.015) compared to euglycemia. However, hyperglycemia-CoCl_2_ led to decrease in VEGFR2 at 12 h of CoCl_2_ (−1.4-fold, *p* = 0.034) compared to hyperglycemia (Figure S3b). 

#### 2.7.1. MAPK Pathway; Metformin Increases Phosphor-ERK1/2 to Total ERK Ratio under Hyperglycemia-CoCl_2_

**Figure 5:**
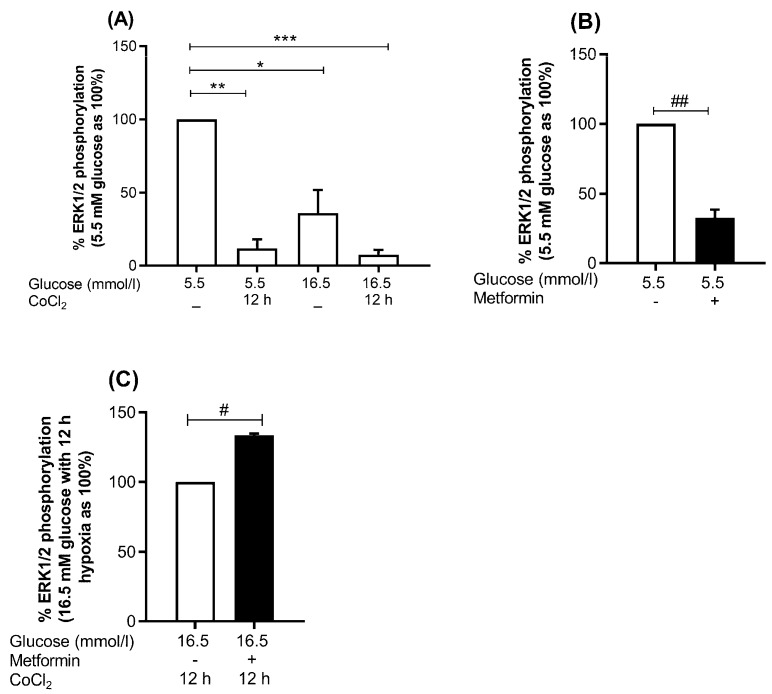
Effect of metformin on the activity of MAPK pathway. HUVEC were treated with euglycemia or hyperglycemia in the presence or absence of metformin for 48 h, and parallel cultures were exposed to CoCl_2_ for 12 h. (**A**) Represents % of ERK1/2 phosphorylation (**B**) Effect of metformin on euglycemia, and (**C**) hyperglycemia combined with 12 h chemical hypoxia CoCl_2_. Percentage of phosphorylation indicates relative intensity of phospho-protein/total protein. The variation in protein expression levels was assessed by flow cytometry on three independent biological replicates. Results are presented as mean ± SEM and were analyzed using paired *t*-test. * *p* < 0.05, ** p < 0.01, *** p < 0.002 compared to the control. ^#^ *p* < 0.05, ^##^ *p* < 0.01 compared pairwise, i.e., metformin-treated condition versus untreated condition.

Metformin led to significant decrease of phospho-ERK1/2 to total ERK ratio to 32.5% (*p* = 0.007) under euglycemia-normoxia (Figure 5B). Whereas an increase of phopho-ERK1/2 to total ERK ratio by 33.6% (*p* = 0.024) was observed under hyperglycemia-CoCl_2_ at 12 h of CoCl_2_ (Figure 5C). No change in the activity of ERK1/2 was detected under euglycemia-CoCl_2_ or hyperglycemia-normoxia when compared pairwise.

We observed a significant decrease in phosphorylation ratio of ERK1/2 to 12.0% (*p* = 0.004) at 12 h of CoCl_2_ under euglycemia, to 33.8% (*p* = 0.05) under hyperglycemia alone, and to 7.5% (*p* = 0.001) at 12 h of CoCl_2_ under hyperglycemia-CoCl_2_ (Figure 5A). Whereas, no significant change in the expression of total ERK1/2 protein was detected in all conditions assayed.

#### 2.7.2. Metformin Increases the Expression of *CXCL8*, *LY96*, *ROCK1*, *MMP16*, *TFPI2*, and *FABP4* under Hyperglycemia-CoCl_2_

Microarray data showed that metformin, under hyperglycemia-CoCl_2_ at 12 h of CoCl_2_, significantly upregulated chemokine (C-X-C Motif) ligand 8 (*CXCL8*) to 1.9-fold (*p* = 2.84 × 10^−2^), lymphocyte antigen 96 (*LY96*) to 1.6-fold (*p* = 2.01 × 10^−3^), *MMP16* to 1.7-fold (*p* = 2.34 × 10^−2^), and Rho-associated, coiled-coil containing protein kinase 1 (*ROCK1*) to 2.7-fold (*p* = 1.15 × 10^−2^) that are known to enhance cell migration. Furthermore, metformin led to a significant overexpression of tissue factor pathway inhibitor 2 (*TFPI2*), a regulator of EC migration [6], to 1.6-fold (*p* = 2.07 × 10^−4^) under hyperglycemia-CoCl_2_ at 12 h of CoCl_2_.Whilst, this effect was not observed without metformin leading to downregulation of *LY96* to −1.7-fold (*p* = 5.4 × 10^−4^), and *TFPI2* to −1.7-fold (*p* = 1.4 × 10^−5^). The most differentially expressed genes affected by metformin under hyperglycemia-CoCl_2_ are summarized in Table 1.

Fatty acid binding protein 4 (*FABP4*) mRNA levels were downregulated at 12 h of CoCl_2_ in euglycemia (−1.6-fold, *p* = 2.4 × 10^−2^) and hyperglycemia (−1.6-fold, *p* = 2.9 × 10^−2^). Conversely, the expression of *FABP4* was significantly induced by adding metformin to HUVEC cultured under hyperglycemia-CoCl_2_ at 12 h of CoCl_2_ (1.6-fold, *p* = 2.53 × 10^−2^).

**Figure 6:**
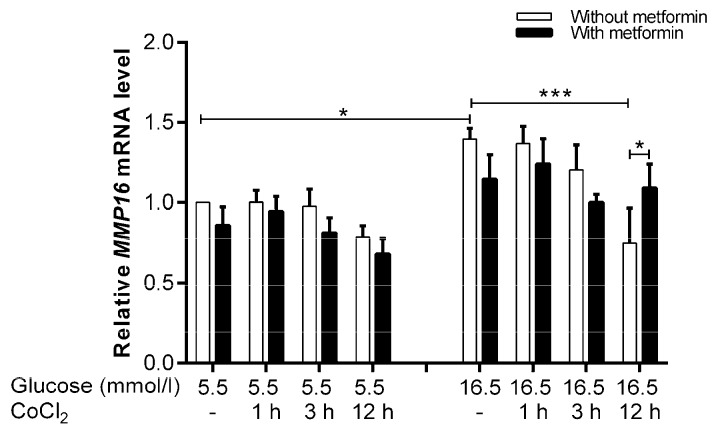
Effect of metformin on *MMP16* mRNA expression in euglycemic and hyperglycemic conditions with CoCl_2_ induction. The variation in RNA expression levels of *MMP16* was assessed by qRT-PCR on three independent biological replicates. Results are presented as mean ± SEM and were analyzed using one-way ANOVA followed by LSD. * *p* < 0.05, *** p < 0.001 compared to the control or hyperglycemia or pairwise, i.e., metformin-treated condition versus untreated condition.

*MMP16* was selected to be validated by qRT-PCR showing that hyperglycemia increased the mRNA level (1.4-fold, *p* = 0.023) compared to control. There was a decrease in *MMP16* expression under hyperglycemia-CoCl_2_ at 12 h of CoCl_2_ (−1.9-fold, *p* < 0.001) compared to hyperglycemia alone (Figure 6). However, metformin significantly increased the mRNA level of *MMP16* by 1.5-fold, *p* = 0.04 under hyperglycemia-CoCl_2_ at 12 h of CoCl_2_ versus parallel conditions without metformin.

### 2.8. Inhibition of Metformin Action Mediated by MMP16 in Cell Migration Assay

**Figure 7:**
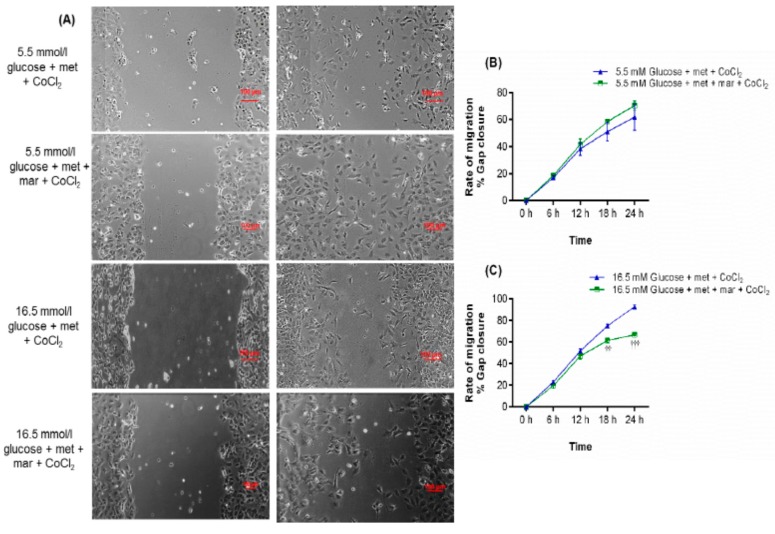
Marimastat antagonizes the effect of metformin on cell migration in HUVEC exposed to hyperglycemia-CoCl_2_. (**A**) HUVEC were incubated with euglycemia or hyperglycemia in the presence or absence of metformin for 24 h. Images were acquired every hour, and three independent biological experiments were performed in duplicate for each condition. The scratch area was measured using NIS Elements software. (**B**) Marimastat had no significant effect on cells treated with euglycemia, metformin and CoCl_2_ induction. (**C**) The effect of metformin was inhibited by marimastat treatment. Results are expressed as mean ± SEM and were analyzed using one-way ANOVA followed by LSD, ^††^ *p* < 0.01, ^†††^ *p* < 0.001 compared with marimastat treated versus untreated-condition. Scale bar is 100 µm. Key: mar: marimastat; met: metformin.

To confirm that MMP16 mediates the effect of metformin on cell migration, we used an MMP16 antagonist, marimastat. Treatment with marimastat reversed the positive effect of metformin on cell migration at 18h (*p* < 0.01) and 24 h (*p* < 0.001) as shown in Figure 7A,C. As metformin had no effect on VEGF signaling in HUVEC exposed to euglycemia-CoCl_2_, marimastat had no effect on cell migration of ECs treated with metformin under euglycemia-CoCl_2_ versus parallel conditions without metformin (Figure 7B).

## 3. Discussion

Large randomized controlled trial has shown that metformin improves CVD outcome in diabetic patients [6]. The current study was aimed to understand the mechanism behind the action of metformin, in order to facilitate the identification of novel markers and therapeutic targets to reduce macrovascular disease. Therefore, we report the effect of metformin on cell survival and migration under conditions of acute hyperglycemia and hyperglycemia-chemical hypoxia combined, with the focus on important VEGF pathway targets. Furthermore, to keep our research relevant to clinical practice, we specifically avoided supra-physiological doses of metformin and used concentrations as anticipated in patients treated with metformin [12]. In addition, we have used relevant hyperglycemia (16.5 mmol/L) which can be observed in diabetic patients as opposed to extremely high glucose levels that are taken up by numerous studies [14,15]. This glucose concentration was also high enough to cause inflammatory changes without causing cytotoxicity [16,17].

Impaired angiogenesis in response to ischemia is elaborated as major macrovascular complications in DM [18]. Additionally, previous study on streptozotocin-induced diabetic mice showed impaired cell migration and tube formation which was improved by metformin [19]. This enhanced effect was attributed to increased expression of phosphorylated-AMP-activated protein kinase, phosphorylated- endothelial nitric oxide synthase and augmented nitric oxide production by metformin in endothelial cells of diabetic mice. For the first time, we have shown that physiological concentrations of metformin improved EC survival and migration under hyperglycemia-chemical hypoxia through activation of VEGF signaling pathway. In our study, an exposure of EC to hyperglycemia lead to a marked increase in cell migration when compared to euglycemia and reduction in cell migration when treated with hyperglycemia-CoCl_2_, as reported previously [20]. Furthermore, we have shown that metformin inhibited cell migration under euglycemia-CoCl_2_ while improving migration under hyperglycemia-CoCl_2_. Esfahanian et al., have reported a similar inhibiting effect of metformin in EC migration under euglycemia although at supra-physiological metformin (0.5–3.0 mmol/L) concentrations [21].

Vascular remodeling by cell migration and angiogenesis is regulated mainly by VEGF signaling [22]. It has been established by others that VEGFA production is regulated by HIF which supports an early involvement of VEGF in angiogenesis [23]. As a downstream of HIF, VEGF induction by hypoxia is delayed with maximal levels at 48 h as reported by others [24,25]. Metformin’s effect on VEGFA165 protein is short lived under hyperglycemia-CoCl_2_ (1 h), and this may be beneficial as a protective mechanism against prolonged angiogenesis. For instance, 1 h of chemical hypoxia is a critical time defined in clinical practice when patients benefit from cardiovascular intervention following acute myocardial infarction.

Our microarray experiments demonstrated that metformin upregulated downstream targets in VEGFA pathway; *MMP16*, *FABP4*, *ROCK1*, *TFPI2*, *CXCL-8*, and *LY96* under hyperglycemia-CoCl_2_, which play a part in cell migration. The role of metformin on migration was confirmed in our experiments using VEGF and MMPs inhibitors. *MMP16* is a membrane-type MT3-MMP that contributes to angiogenesis by degrading extracellular matrix components, thus promoting cell migration and bioavailability of growth factors [26,27]. Park et al., showed that upon secretion, VEGF becomes bound to the extracellular matrix (ECM) and acts in a paracrine fashion [28]. The interaction of VEGF with matrix proteins is mediated through the carboxy-terminal region, also known as an ECM-binding domain [29,30]. The regulation of VEGF in the extracellular environment has also been implicated in the angiogenic switch [31]. A prior study found that inhibition of MMP16 significantly reduces the VEGFA-enhanced tube formation in ECs [32]. This is in agreement with our results from the scratch assay where the pro-angiogenic effect of metformin in the combined hyperglycemia-CoCl_2_ treatment was inhibited by marimastat, an MMP inhibitor.

A downstream mediator of VEGF/VEGFR2 in our study, *FABP4* [33] was found to be increased by metformin under hyperglycemia-CoCl_2_ at 12 h of CoCl_2_ enhancing cell survival and migration. FABP4 knockdown in HUVEC has been shown to decrease cell migration and increase apoptosis [33], whilst VEGF knockdown was lethal [34,35]. Additionally, metformin enhanced the expression of *ROCK1* under hyperglycemia-CoCl_2_, another moderator of cell migration. RhoA/ROCK signaling had previously shown to promote endothelial migration in response to VEGFA through the modulation of the actin cytoskeleton organization [36]. Moreover, *ROCK* activation appears to be a key event in the initiation of angiogenic process by mediating an increase in endothelial permeability and migration, whilst ROCK1 inhibitor suppressed cell migration as shown recently [37]. We also found that metformin upregulated the proinflammatory chemokine *CXCL8*. This chemokine revealed pro-angiogenic properties by preserving VEGFA expression and secretion [38], whereas anti-CXCL8 was reported to reduce EC migration [39].

A Kunitz-type serine proteinase inhibitor TFPI-2 [40], upregulated by metformin in our study, was shown to be overexpressed in migrating EC [41]. Previous studies have demonstrated that VEGF mediates the induction of TFPI-2 through ERK1/2 activation [42,43]. However, as TFPI-2 was detected to control cell migration [41], it presumably acts as an important regulator to prevent excessive and uncontrolled cell migration.

Our results demonstrate that metformin inhibited apoptosis under combined hyperglycemia-CoCl_2_ through VEGF and activation of VEGF downstream signaling pathways. This was confirmed by using VEGF inhibitor (sunitinib) in the apoptosis assay. It is known that VEGF activates the phosphorylation of ERK1/ERK2 leading to activation of MAPK pathway, which is an essential step in determining the survival of EC [44]. We found that the activity of the ERK/MAPK pathway was inhibited by euglycemia-CoCl_2_, hyperglycemia, and hyperglycemia combined with CoCl_2_ at 12 h which is measured by MAPK activation dual detection assay. A previous study demonstrated that the level of phospho-ERK1/2 in astrocytes was elevated after 1 h ischemia and reached a maximal level after 4 h ischemia, before decreasing at 5 h resulting in enhanced ischemia-induced cell death [45]. In another study activation of ERK/MAPK signaling was required for cardioprotection from ischemia-reperfusion injury in vivo through antagonism of apoptotic regulatory pathways [46]. In our experiments we were able to demonstrate that metformin activates ERK/MAPK signaling in HUVEC exposed to hyperglycemia-CoCl_2_, augmenting cell survival. Therefore, metformin may have properties to enhance cardioprotection from ischemia-reperfusion; however, we have not studied the reperfusion phase in our study.

Previous studies have shown that VEGFA expression was significantly elevated in diabetic patients with chronic coronary heart disease, and the cellular response to VEGFA was attenuated due to defect in VEGF receptors and downstream signal transduction [47,48]. Our data confirm these findings of overexpression of VEGFA combined with downregulation of VEGFR2 and VEGF downstream intracellular signaling in HUVEC exposed to hyperglycemia-CoCl_2_. 

**Figure 8:**
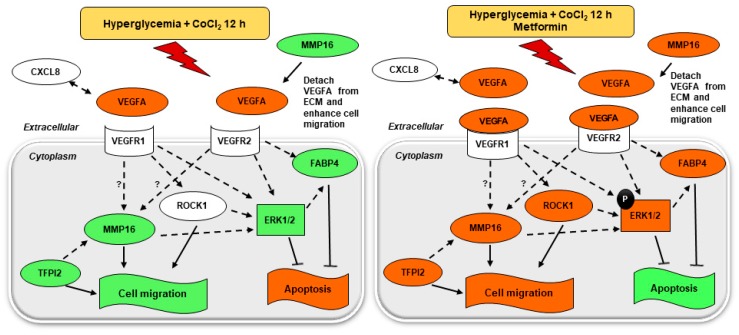
Comprehensive VEGF signaling network of genes and proteins involved in cell migration and survival. Metformin-treated condition is compared to metformin-untreated condition under hyperglycemia-CoCl_2_ for 12 h. The network was created by IPA software rendering VEGF signal transduction pathways. The genes from microarray expression study that are represented with red shades are upregulated, and green shades are downregulated, *MMP16* was validated by qRT-PCR. The activity of ERK1/2 was assessed by MAPK activation dual detection assay flow cytometry. The red shade on functional assays denoted activation while green shade inhibition. Solid lines denoted direct interaction; interrupted lines denoted indirect interaction.

Thus, the pivotal effect of metformin as documented by us was to restore the VEGFA pathway to non-diabetic state by augmenting the expression of VEGFR1 and VEGFR2 that transduce the vasoprotective downstream signalings of VEGF (Figure 8); a mechanism also documented in other cardioprotective therapies such as statins (HMG CoA reductase inhibitors) [49]. The lack of metformin’s beneficial effect in euglycemia is related to the fact that the VEGF related genes were principally unaltered in euglycemia-CoCl_2_. In line with these findings, clinical studies confirm that metformin offers no benefit in cardiovascular outcome in non-diabetic individuals [50,51]. Our data shows that metformin has a direct effect on several targets within VEGFA pathway and therefore could lead to further drug discovery of other cardioprotective therapies.

## 4. Materials and Methods

### 4.1. HUVEC Cultures

The study was approved by the Biomedical Ethics Unit, Faculty of Medicine, King Abdulaziz University (approval number: 440-10) and NRES Committee North East-Sunderland, UK (approval number: 12/NE/0044). HUVEC were harvested from three independent umbilical cords as previously described [17]. All experiments were performed in passage two. HUVEC were incubated in a culture medium with 5.5 mmol/L (euglycemia) or 16.5 mmol/L (hyperglycemia) glucose concentrations (Sigma-Aldrich, Dorset, UK) for 24 h except for functional assays (scratch, apoptosis, MAPK) for 48 h [16,17] and in presence/absence of 0.01 mmol/L metformin (Sigma-Aldrich) for up to 24 h or 48 h. Subsequently, HUVEC were incubated under normoxia or CoCl_2_ for 1, 3, or 12 h using a final concentration of 150 µmol/L CoCl_2_ (Sigma-Aldrich, Dorset, UK) as per protocol [52] in the same experiment. Whereas up to 24 h CoCl_2_ exposure was utilized in functional assays (scratch assay, apoptosis). Metformin concentration was selected according to the peak plasma concentration suggested by FDA fact sheet [10]. The concentration of metformin used was validated previously as tube formation was increased at 0.005–0.05 mmol/L but decreased at 0.5 mmol/L [53].

### 4.2. In Vitro Scratch Assay (Cell Migration Assay)

HUVEC were subcultured onto 24 well plate (1 × 10^5^ cells/well). Cells were incubated under euglycemia or hyperglycemia in the presence/absence of metformin for 24 h. Scratch lines were created on confluent HUVEC monolayers using 1000 µL pipette tip. The medium was replaced with fresh medium containing respective concentrations of glucose and metformin, and then the cells were exposed to CoCl_2_ for up to 24 h. As a negative control for cell migration, HUVEC were treated with marimastat, an matrix metalloproteinase (MMP) inhibitor (Sigma-Aldrich, Dorset, UK) to a final concentration of 10 µmol/L in DMSO [54] or 0.1 µmol/L sunitinib malate a VEGF inhibitor (Sigma-Aldrich, Dorset, UK) [55]. A subtoxic sunitinib dose was selected based on our previously published results [12]. Subsequently, the cells were incubated in a 5% CO_2_ chamber (OkoLab) for 24 h that was connected to the camera (Hamamatsu Orca ER, Hamamatsu, Japan). Cell migration was examined by phase-contrast microscopy (Nikon Eclipse Tie, Tokyo, Japan) using software-based autofocus (NIS Elements V4.13, Nikon, Tokyo, Japan). Images were acquired every hour, and three independent biological experiments were performed at which each condition was assessed in duplicate. The scratch area in each image was measured using NIS Elements software. Cell migration is presented as a percentage of gap closure and has been calculated by the following equation:

$$The\;percentage\;of\;gap\;closure=\frac{Area\;\left(0\right)-\;Area\;\left(t\right)}{Area\;\left(0\right)}\times \;100$$

where *Area* (0) is the area of scratch pre-migration, *Area* (*t*) is an area of the scratch after migration at time *t*.

### 4.3. Apoptosis Assay

HUVEC were treated in passage two at 60% confluency. The supra-physiological concentration of metformin 1.0 mmol/L on apoptosis was additionally used. Culture media and metformin were replaced every 12 h. A positive control for the apoptosis assay was established by treating the cells with 14 µmol/L sunitinib malate for 24 h. To measure apoptosis, Annexin V APC staining assay (BD, Bioscience, San Jose, CA, USA) was performed according to the manufacturer’s recommendations and acquiring 10,000 events by FACSAria III flow cytometer (BD, Bioscience, San Jose, CA, USA). 

### 4.4. Total RNA Extraction

Total RNA from HUVEC was extracted using the RNeasy Mini Kit (QIAGEN, Hilden, Germany) and on-column DNase digestion with RNase-Free DNase set (QIAGEN) was performed according to the manufacturer’s instructions. The integrity of RNA samples was assessed by using Agilent 2100 Bioanalyzer (Santa Clara, CA, USA) yielding high RNA Integrity Numbers (RIN) between 9.1 and 10. 

### 4.5. Microarray Experiments and Gene Expression Analysis

Microarray experiments were performed using Affymetrix (Santa Clara, CA, USA) Human Gene 1.0 ST arrays according to manufacturer’s instructions with minor modifications [17,56]. Three biological replicates were hybridized for each experimental condition resulting in a total of 48 microarray experiments.

Affymetrix CEL files were imported into Partek Genomic Suite version 6.6 (Partek Inc., Chesterfield, MO, USA) and then data were normalized and analyzed as described previously [12]. The complete dataset and associated experimental information were submitted to NCBI’s Gene Expression Omnibus with accession number GSE46263. Ingenuity pathway analysis (IPA) software version 9 (Ingenuity, Redwood City, CA, USA) was used to explore the Canonical Pathways that may be activated or inhibited. Additionally, IPA assisted in detecting the interactive molecular and cellular functions in each condition.

### 4.6. Quantitative RT-PCR

Total RNA (100 ng) from each sample was reversed transcribed to cDNA using SuperScript^®^ VILO™ cDNA Synthesis Kit (Life Technologies, Paisley, UK) in a final volume of 20 μL. cDNA were quantified by hydrolysis probe real-time PCR performed with TaqMan^®^ Universal Master Mix II (Life Technologies) using StepOne Plus Real-time PCR system (Life Technologies) according to the manufacturer’s recommendations. Expression of the selected genes *VEGFA* and *MMP16* was validated using TaqMan gene expression assays (*VEGFA* ID Hs0090005_m1, *MMP16* ID Hs0023467_m1, Life Technologies). Three independent biological replicates were tested for each gene, and all samples were run in triplicates. The Comparative *C*_t_ (ΔΔ*C*_t_) method was applied to the mean value of each triplicate to quantify expression of the target genes, which were normalized to reference control gene for the large ribosomal protein P0 [17] (Life Technologies).

### 4.7. Protein Studies

Protein was extracted using the mirVana PARIS (Ambion, Grand Island, NY, USA) and the concentration was measured using Pierce Microplate BCA protein assay (Thermo Scientific, Waltham, MA, USA) according to the manufacturer’s protocol. The concentration was scanned using ELISA plate reader (Multiskan Ascent, Thermo Labsystems, Waltham, MA, USA). Western blots were performed using commercially available kits (Life Technologies) XCell SureLock (Life Technologies) for protein fractionation, and Mini Protean II (BioRad, Hercules, CA, USA) for blot transfer. Nitrocellulose membranes (Life Technologies) were blocked with blocking buffer from Li-COR (Lincoln, NE, USA) and blocking buffer was diluted 1:2 with 1× TBS-T for incubation with antibodies. Transferred protein were probed for hypoxia-inducible factor-1α (HIF-1α, mouse monoclonal anti-HIF-1α, 5:3000; Abcam, Cambridge, UK), VEGFA (mouse monoclonal anti-VEGFA, 1:1000; Abcam), and VEGF165A (mouse monoclonal anti-VEGF 165A, 1:1000; Abcam) and subsequently a secondary antibody (IRDye^®^ 680RD goat (polyclonal) anti-mouse IgG, 0.5:5000; Li-COR) was applied. β-actin (mouse monoclonal anti-beta actin, 1:3000; Abcam) was used as a loading control. Anti-VEGF showed many extra protein non-specific bands; therefore, we studied VEGF165A in detail. Bands were detected by infrared imager Odyssey (Li-COR) and quantified by Image Studio Lite version 3.1 (Li-COR Biosciences, Lincoln, NE, USA). For stripping, bound antibodies were removed by incubation in Restore^TM^ Plus Western Blot Stripping Buffer (Thermo Scientific, Waltham, MA, USA). 

VEGFR1/Flt-1 (R & D Systems, Minneapolis, MN, USA), VEGFR-2 and phospho-VEGFR-2 (Cell Signaling, Danvers, MA, USA) were assessed by ELISA according to the manufacturers’ instructions. 

### 4.8. MAPK Activation Dual Detection Assay

The activation of mitogen-activated protein kinase (MAPK) signaling pathway was measured by phosphorylation of extracellular signal-regulated protein kinases 1 and 2 (ERK1/2) using the FlowCellect™ MAPK Activation Dual Detection kit (Millipore, Darmstadt, Germany) which includes two antibodies, a phospho-specific anti-phospho-ERK1/2 (Thr202/Tyr204, Thr185/Tyr187)-PE and, for quantification of total levels of ERK, an anti-ERK1/2-Alexa Fluor^®^ 647 conjugated antibody. HUVEC were treated with 5.5 mmol/L) or 16.5 mmol/L glucose concentrations in the presence/absence of metformin (0.01 mmol/L) for 48 h and then were exposed to CoCl_2_ for 12 h. All conditions were assayed in 2 or 3 independent replicates. Culture media and metformin were replaced every 12 h. For VEGF inhibitor assays, cells were incubated with EBM-2 medium containing VEGF as a positive control or with EBM-2 medium containing 7 µmol/L sunitinib malate as a negative control. Subsequently, 2 × 10^5^ cells were fixed by an equal volume of ice-cold fixation buffer for 20 min and processed according to the manufacturer’s instructions by acquiring 14,000 events using FACSAria III flow cytometer.

### 4.9. Statistical Analysis

Results are presented as mean ± SEM. Statistical analysis was performed using one-way ANOVA followed by post-hoc analysis Fisher’s least significant difference (LSD) test for qRT-PCR, ELISA, western blot, and in vitro scratch assays. The Student’s *t*-test between any two experimental groups was applied for the MAPK activation dual detection assay and Annexin V Apoptosis assay. Calculations were performed using IBM SPSS software version 21.0 (SPSS Inc., Armonk, NY, USA). A *p*-value < 0.05 was considered statistically significant.

## 5. Conclusions

In conclusion, our in vitro study demonstrates that the effect of metformin in acute hyperglycemia-chemical hypoxia model is mediated through restoring VEGFA pathway. This occurs through upregulation of VEGFR1 and VEGFR2, leading to dual effect on activation of cell migration through *MMP16* and *ROCK1* upregulation, in addition to inhibition of apoptosis by increase in phospho-ERK1/2 and *FABP4*, components of VEGF signaling cascades.

## Figures and Tables

**Table 1:** The top differentially expressed genes in HUVEC treated with metformin and exposed to hyperglyemia-CoCl_2_.

Gene Name	Gene Symbol	*p*-Value	FC Hyperglycemia-CoCl_2_ 3 h + Metformin	*p*-Value	FC Hyperglycemia-CoCl_2_ 12 h + Metformin
small nucleolar RNA, H/ACA box 20	*SNORA20*	5.45 × 10^−1^	−1.28	1.23 × 10^−3^	4.01
small nucleolar RNA, C/D box 45C	*SNORD45C*	3.62 × 10^−1^	−1.50	1.26 × 10^−2^	3.16
metastasis associated lung adenocarcinoma transcript 1 (non-protein)	*MALAT1*	2.88 × 10^−2^	2.38	8.78 × 10^−3^	2.87
Rho-associated, coiled-coil containing protein kinase 1	*ROCK1*	9.38 × 10^−2^	1.90	1.15 × 10^−2^	2.68
cerebellar degeneration-related protein 1, 34 kDa	*CDR1*	5.47 × 10^−3^	2.80	1.20 × 10^−2^	2.52
Chemokine (C-X-C Motif) ligand 8	*CXCL8*	1.49 × 10^−1^	1.54	2.84 × 10^−2^	1.94
cytochrome c oxidase subunit VIIb	*COX7B*	4.23 × 10^−1^	1.17	5.53 × 10^−3^	1.76
matrix metallopeptidase 16 (membrane-inserted)	*MMP16*	8.78 × 10^−1^	−1.04	2.34 × 10^−2^	1.73
fatty acid binding protein 4	*FABP4*	2.92 × 10^−2^	1.58	2.53 × 10^−2^	1.60
lymphocyte antigen 96	*LY96*	2.29 × 10^−1^	1.18	2.01 × 10^−3^	1.57
tissue factor pathway inhibitor 2	*TFPI2*	6.17 × 10^−1^	1.06	2.07 × 10^−4^	1.55

HUVEC were incubated with high glucose concentration 16.5 mmol/L and 0.01 mmol/L metformin then chemical hypoxia was induced by 150 µmol/L CoCl_2_. Affymetrix .CEL files were imported to Partek Genomic Suite version 6.6 and normalized using RMA. Differentially expressed gene sets were generated using one-way ANOVA, FDR-unadjusted *p*-value < 0.05 and fold change (FC) cutoff of 1.5 compared pairwise, i.e., the condition treated with and without metformin. Subsequently, the most influenced genes were compared among different conditions of hypoxia. The signs in the FC column denote (−) downregulated, (+) upregulated genes.

## Supplementary Materials

Supplementary materials can be found at www.mdpi.com/1422-0067/19/1/293/s1.

## Abbreviations

CVD	Cardiovascular disease
CXCL8	Chemokine (C-X-C Motif) Ligand 8
DM	Diabetes mellitus
EC	Endothelial cell
ERK1/2	Extracellular signal-regulated protein kinases 1 and 2
FABP4	Fatty acid binding protein 4
LY96	Lymphocyte antigen 96
MAPK	Mitogen-activated protein kinase
MI	Myocardial infarction
MMP16	Matrix metalloproteinase 16
PI3K	Phosphatidylinositol 3′-kinase
ROCK1	Rho-associated, coiled-coil containing protein kinase 1
TFPI2	Tissue factor pathway inhibitor 2
VEGFA	Vascular endothelial growth factor A
