# Supplementary material for: Proangiogenic Effect of Metformin in Endothelial Cells Is via Upregulation of VEGFR1/2 and Their Signaling under Hyperglycemia-Hypoxia
Source: Int J Mol Sci. 2018 Jan 19;19(1):293. doi: 10.3390/ijms19010293 (PMC5796238; doi:10.3390/ijms19010293)
Supplement: Supplementary file 1 [file ijms-19-00293-s001.pdf]

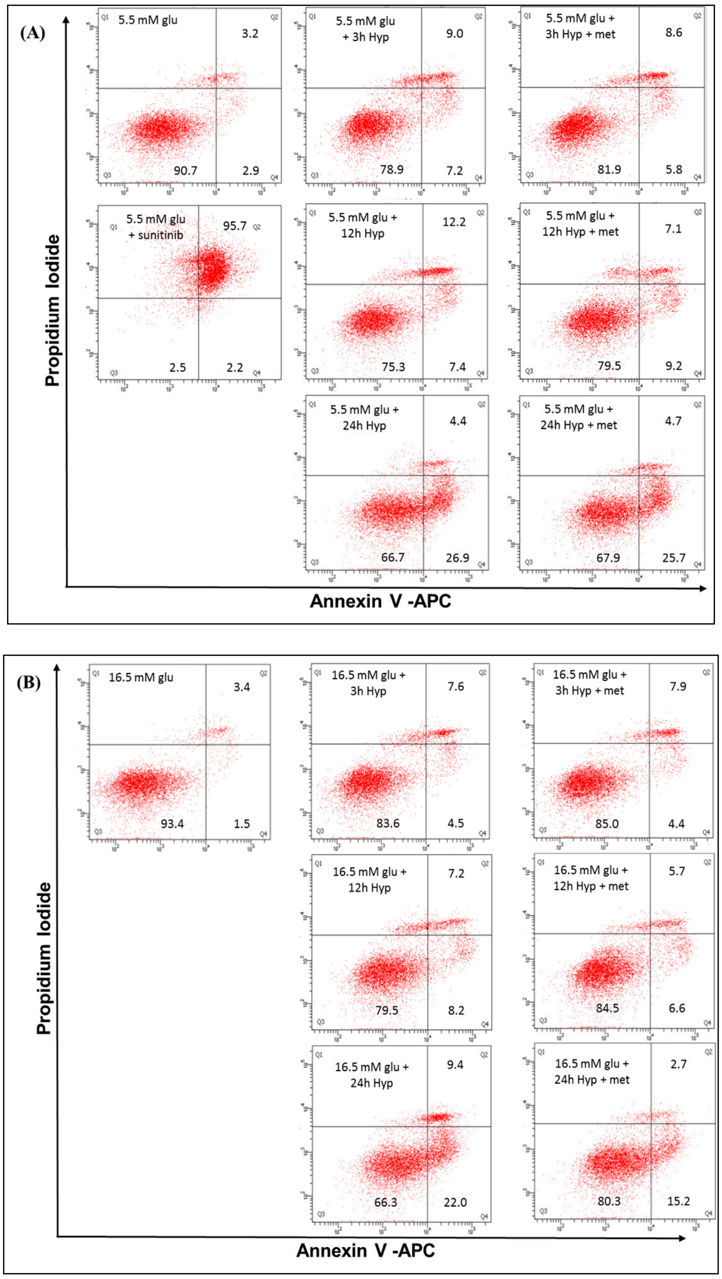

Figure S1. Representative dot plots of Annexin V APC staining assay for apoptosis. HUVEC were treated with (**A**) 5.5 mmol/L or (**B**) 16.5 mmol/L for 48 hours in the presence or absence of physiological metformin concentration, and parallel cultures were exposed to chemical hypoxia for 3, 12 or 24 hours. Key: glu: glucose; hyp: hypoxia; met: metformin.

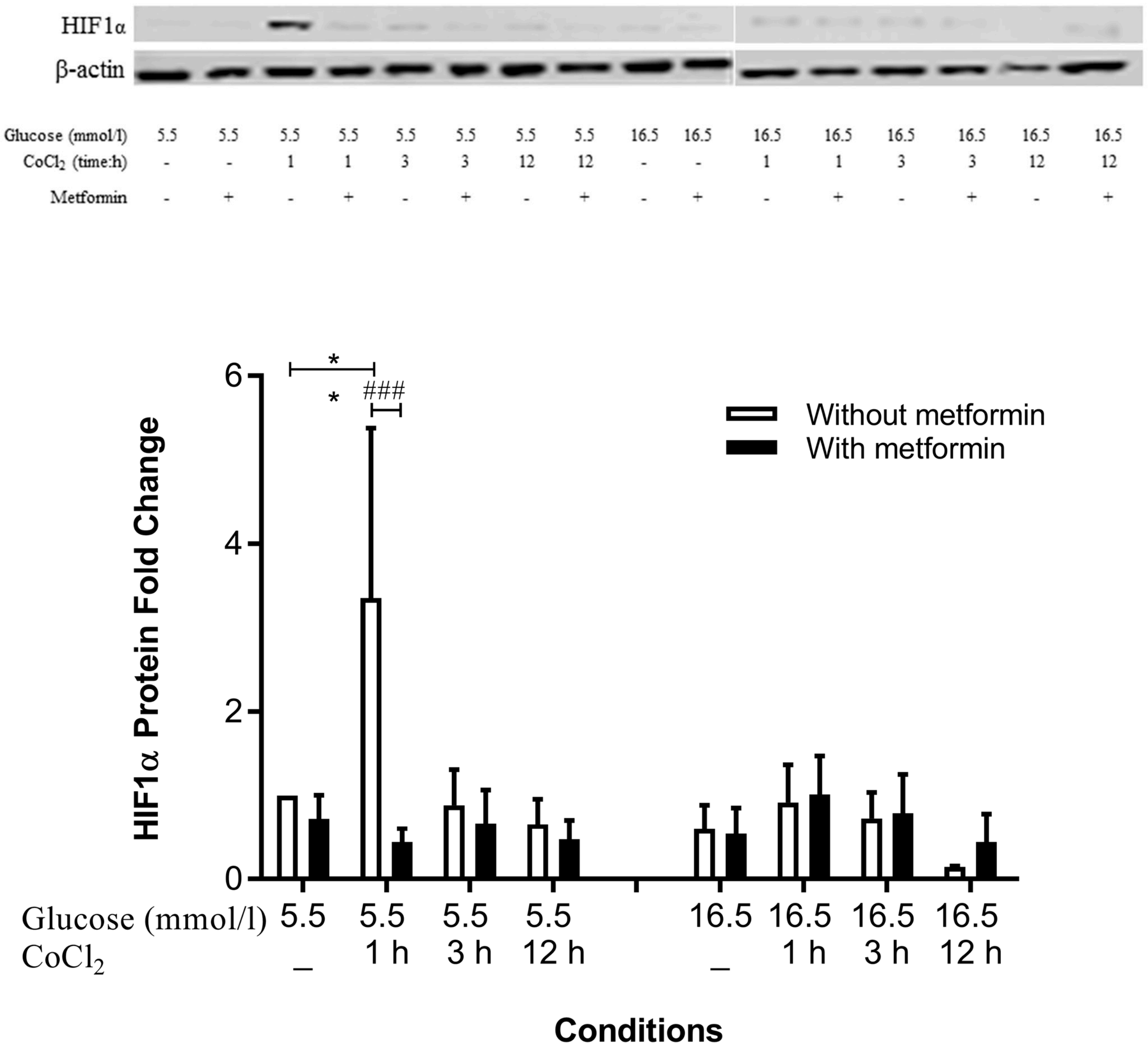

Figure S2. Effect of metformin on HIF-1α protein expression in euglycemic and hyperglycaemic conditions with chemical hypoxia. HUVEC were treated with hyperglycaemic (16.5 mmol/L) or euglycemic (5.5 mmol/L) glucose concentrations. After 24 hours, metformin (0.01 mmol/L) was added to euglycemic and hyperglycaemic cultures and then exposed to $CoCl_2$ for either 1, 3 or 12 hours. The variation in protein expression levels of HIF-1α was assessed by Western blot from three independent biological replicates. Results are presented as the mean ± SEM and were analysed using one-way ANOVA followed by the LSD test. $^{**}p < 0.01$ compared to the control. $^{###}p < 0.001$ compared pairwise, without metformin versus metformin-treated conditions

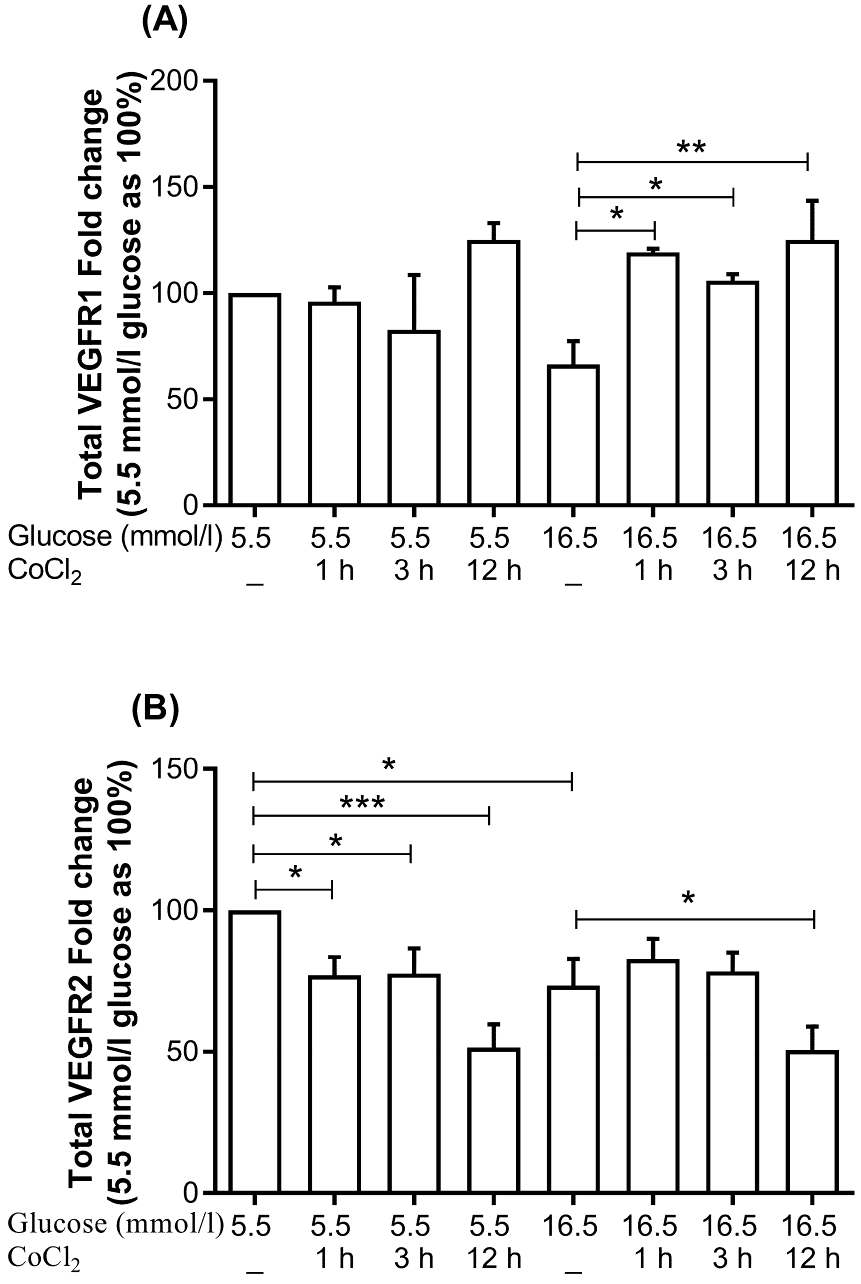

Figure S3. Effect of hypoxia on VEGF receptors. HUVEC were treated with hyperglycaemic (16.5 mmol/L) or euglycemic (5.5 mmol/L) glucose concentrations. After 24 hours, chemical hypoxia was induced for either 1, 3 or 12 hours. The variation in protein expression levels of VEGFR1 and VEGFR2 was assessed by ELISA from three independent biological replicates. Results are presented as the mean ± SEM and were analysed using one-way ANOVA followed by the LSD test. $^{*}p < 0.05$, $^{**}p < 0.01$, $^{***}p < 0.001$ compared to the control.

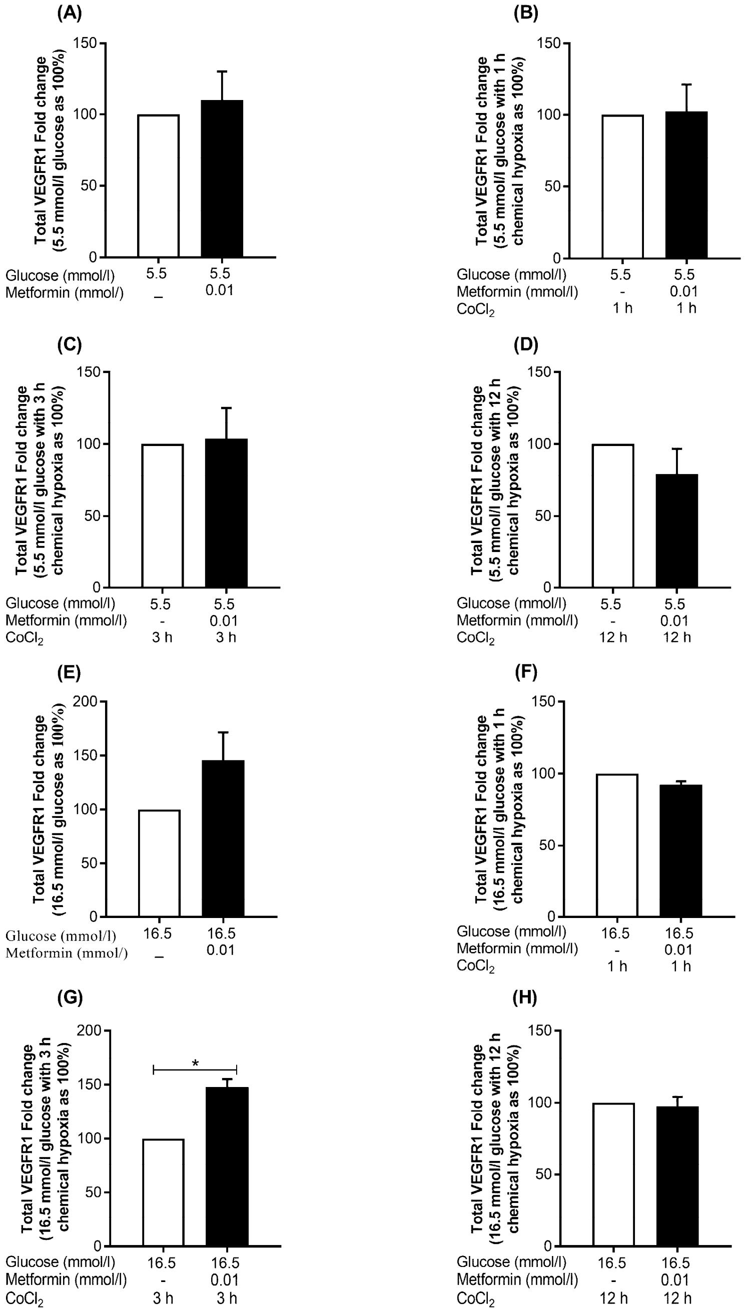

Figure S4. Effect of metformin on the protein expression of VEGFR1. HUVEC were treated with euglycemia or hyperglycemia in the presence or absence of physiological metformin concentration (0.01 mmol/l) for 24 h, and parallel cultures were exposed to chemical hypoxia (150 µmol/l $CoCl_2$). The variation in protein expression levels of VEGFR1 was assessed by ELISA from three independent biological replicates. (**A**) Effect of metformin on euglycemia glucose, (**B**) euglycemia combined with 1 h $CoCl_2$ exposure, (**C**) euglycemia combined with 3 h $CoCl_2$ exposure, (**D**) euglycemia combined with 12 h $CoCl_2$ exposure, (**E**) hyperglycemia, (**F**) hyperglycemia combined with 1 h $CoCl_2$ exposure, (**G**) hyperglycemia combined with 3 h $CoCl_2$ exposure and (**H**) hyperglycemia combined with 12 h $CoCl_2$ exposure. Results are presented as the mean ± SEM and were analysed using the paired *t*-test. $^{*}p < 0.05$ compared pairwise, the conditions treated with versus without metformin

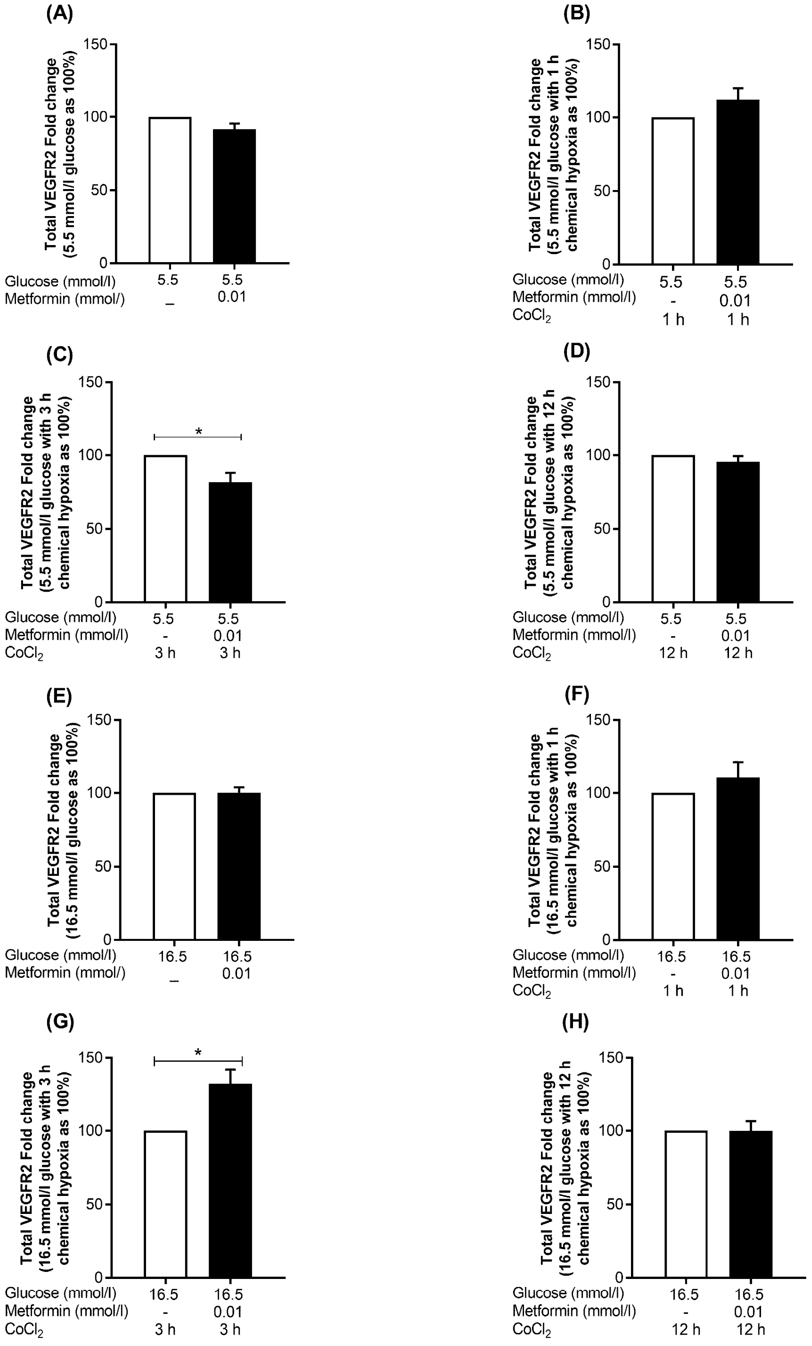

Figure S5. Effect of metformin on the protein expression of VEGFR2. HUVEC were treated with euglycemia or hyperglycemia in the presence or absence of physiological metformin concentration (0.01 mmol/L) for 24 h, and parallel cultures were exposed to chemical hypoxia. The variation in protein expression levels of VEGFR2 was assessed by ELISA from three independent biological replicates. (**A**) Effect of metformin on euglycemia glucose, (**B**) euglycemia combined with 1 h $CoCl_2$ exposure, (**C**) euglycemia combined with 3 h $CoCl_2$ exposure, (**D**) euglycemia combined with 12 h $CoCl_2$ exposure, (**E**) hyperglycemia, (**F**) hyperglycemia combined with 1 h $CoCl_2$ exposure, (**G**) hyperglycemia combined with 3 h $CoCl_2$ exposure and (**H**) hyperglycaemia combined with 12 h $CoCl_2$ exposure. Results are presented as the mean ± SEM and were analysed using the paired *t*-test. $^*p < 0.05$ compared pairwise, the conditions treated with versus without metformin
